# A Retrospective Study of Causes of Low Vision in Saud Arabia, A Case of Eye World Medical Complex in Riyadh

**DOI:** 10.5539/gjhs.v8n5p205

**Published:** 2015-10-20

**Authors:** Abdullah Z. Alotaibi

**Affiliations:** 1 Department of Optometry, College of Applied Medical Sciences, King Saud University, Riyadh, Saudi Arabia

**Keywords:** low vision, Saudi Arabia, low vision aids, awareness

## Abstract

Vision is the ability of seeing with a definite understanding of features, color and contrast, and to distinguish between objects visually. In the year 1999, the World Health Organization (WHO) and the International Agency for the Prevention of Blindness formulated a worldwide project for the eradication of preventable loss of sight with the subject of “Vision 2020: the Right to Sight”. This global program aims to eradicate preventable loss of sight by the year 2020. This study was conducted to determine the main causes of low vision in Saudi Arabia and also to assess their visual improvement after using low vision aids (LVD). The study is a retrospective study and was conducted in low vision clinic at Eye World Medical Complex in Riyadh, Saudi Arabia. The file medical record of 280 patients attending low vision clinics from February 2008 to June 2010 was included. A data sheet was filled which include: age, gender, cause of low vision, unassisted visual acuity for long distances and short distances, low vision devices needed for long distances and short distances that provides best visual acuity. The result shows that the main cause of low vision was Optic atrophy (28.9%). Retinitis pigmentosa was the second cause of low vision, accounting for 73 patients (26%) followed by Diabetic retinopathy and Macular degeneration with 44 patients (15.7%) and 16 patients (5.7%) respectively. Inter family marriage could be one of the main causes of low vision. Public awareness should be embarked on for enlightenment on ocular diseases result in consanguineous marriage. Also, it is an important issue to start establishing low vision clinics in order to improve the situation.

## 1. Introduction

### 1.1 Background of the Study

Vision is the capability of seeing with an unambiguous sensitivity of features, color and contrast, and to differentiate between objects visually. Like the other human senses, vision tends to worsen or reduce with age and it is supposed to be a natural phenomenon (Eskridge, Amos, & Bartlett, 1991). In most of the cases, the diminution in the visual functionality can be improved with visual aids usually simple display glasses, contact lenses, medication and sometimes even through surgery. However, if the vision related medical condition occurs because of an untreatable eye infection, circumstance or injury or due to some vision loss than the eye related medical issue could be permanent and insolvable. Visual impairment is one of the important and relatively common issues for older people (Pollard et al., 2003).

In the year 1999, the World Health Organization (WHO) and the International Agency for the Prevention of Blindness formulated a worldwide project for the eradication of preventable loss of sight with the subject of “Vision 2020: the Right to Sight”. This global program aims to eradicate preventable loss of sight by the year 2020, so that the prevalence of the vision related medical condition could be controlled in the future so that all the people in the world can enjoy their right to see the beautiful colors of the world. The project also raises public awareness about the medical conditions associated with blindness & vision impairment as one of the most important worldwide community health issues, so that it would pressurize the Governments around the world and the Ministers of Health of these governments to contribute to the cause and assign funds for nationwide sightlessness deterrence programs it would also educate target audiences about loss of sight avoidance and about VISION 2020 and to create support for VISION 2020 program activities.

In term of ocular diseases, one of the main goals of the initiative is elimination of unnecessary blindness due to some of the basic diseases which are the following diseases or medical conditions: trachoma, Onchocerciasis, cataract and refractive errors. These conditions were specifically selected and targeted in this study not just because of the prevalence of the visual injuries but also due to the possibility and affordability of intervention procedures accessible for the management and avoidance of the disease. Cataract and refractive errors have high frequency of prevalence rate in almost all populations and there are various cost effective vision management interventions. Vision 2020 involves the energetic contribution of the United Nations associated agencies, international governments, worldwide care organizations, healthcare institutions and professionals, charitable institutions and organizations along with the individuals operating jointly in a worldwide affiliation to achieve the objective of world without high prevalence visual medical conditions by the year 2020. It has been documented over the years that foremost efforts and hard work is required to be done and synchronized on a national scale, on the continental or regional scale and on the international scale to reinstate vision to the visually impaired people and to prevent others from losing their sight totally or partially and become blind or visionless.

Globally, a world health organization (WHO) report indicated that there are more or less 285 million inhabitants internationally living with partial or low vision and even with complete loss of sight or blindness. Out of these people, around 39 million people are completely blind or have lost all the abilities to view and about 246 million have partial or moderate or very severe visual impairments. The report shows that, around 90% of the people who are blind or have lost all the ability to see or view are those who live in low income countries or less developed countries of the world. Also, around 65% of the people who have visual impairments are aged over 50 years or even older; however this age group makes up only 20% of the world’s inhabitants which makes this age group as the most highly prevalent age group for visual impairments and blindness.

Visual impairment is not a medical condition which is dispersed consistently all over the globe and has different prevalence rate in different regions like any other medical condition. Majority of the visually impaired people live in the developing countries that is more than 90% of the world’s visually impaired people are living in developing countries (World Health Organization, 2013). It is also a very well documented fact that most of the visual impairments are inoperable and people with low vision are in want of care, this almost certainly means that there is a continuous increase in the low vision clinics throughout the world. In fact, low vision clinics will serve people whose vision cannot be corrected to normal levels through usual means. In other words, the main goal of low vision clinic is to assess a person’s visual capabilities so that appropriate optical devices, ranging from simple hand-held magnifiers to very advanced electronic magnifiers can be prescribed to improve performance on certain tasks such as reading (Hall et al., 1987).

Effective rehabilitation and low vision services can not only improve people’s lives but it would transform people’s lives by serving people to formulate the best possible way to use their residual vision, enabling individuals to lead self-governing and satisfying lives, dropping the figures of falls and accidents connected with visual injury. It has been found that, vision impairment is associated with difficulty in performing everyday livelihood actions, loss of self-determination, falls, social remoteness, despair and depression, impaired physical and psychological health and increase in mortality rate (Pollard et al., 2003; Robbins, 1981). Many authors have mentioned that it is essential that low vision clinics have to expand their range of services to address the psychosocial and non optical needs of people with severely reduced eyesight. Consequently, comprehensive low vision service must be delivered to all low vision patients in order to cover their needs (Hall et al., 1987; Tielsch et al., 1990; Gresset & Baumgarten, 2002).

Many people including old and young all over the globe living with everlasting visual impairment have a number of remaining vision factors that can be utilized with the assistance of low vision services, resources and procedures. While medicinal prevention might not be adequate for individuals with low vision, their excellence of life can be improved to a great extent, and with over 500,000 people suffering from the disabling impairment only in Saudi Arabia, it is surely a very sound and deserving service to provide in order to advance the excellence of life of hundreds and thousands of affected individuals (World Health Organization, 2013).

At the present, a standard low vision service in Riyadh is provided in only two government hospitals that is the King Fahad Medical City (KFMC) and the King Khalid Eye Specialist Hospital (KKESH) along with a private eye centre (Eye World Medical Complex). However, it is not clear or is not certain that whether private eye clinics provide such services with the necessary coordination or not. Thus, this study was conducted to establish the major causes of low vision in Saudi Arabia and also to assess their visual improvement after using low vision aids (LVD).

### 1.2 Objective of the Study

The main goal and objective of the study is elimination of unnecessary blindness through controlling the prevalence of the disease. The study aims to identify the main causes of the visual impairment in the Saudi population so that on the basis of the results, implementable solution and intervention plans could be formulated. This study is conducted to determine the main causes of low vision in Saudi Arabia and also to assess their visual improvement after using low vision aids (LVD).

### 1.3 Hypothesis of the Study

H0: Visual impairment in Saudi Arabia is because of the lack of awareness in the public and the lack of initiative at the government and private level.

H1: Visual impairment in Saudi Arabia is not caused by the lack of awareness in the public or the lack of initiative at the government and private level.

## 2. Methods

### 2.1 Sample Population

This study was conducted in low vision clinic at Eye World Medical Complex in Riyadh, Saudi Arabia. The file medical record of 280 patients attending the low vision clinics from February 2008 to June 2010 was included. Their age was in the range of 5 to 82 years in which 65% of them were male and about 35% of them were female. They were examined by a registered Ophthalmologists and Optometrists during the above mentioned period.

### 2.2 Data Collection and Analysis

A data sheet was filled which include: age, gender, cause of low vision, unassisted visual acuity for long distances and short distances, low vision devices needed for long distances and short distances that provides best visual acuity. The data was analyzed using Statistical Program of SPSS 2.0 and was then evaluated and discussed in the results section.

### 2.3 Research Design and Ethical Considerations

The research design selected for this study is retrospective and the data utilized is secondary data. Since the data utilized is secondary data hence no serious ethical considerations were present however privacy statements were collected from the participants.

## 3. Results

The study included 280 patients. There were 182 (65%) male and 98 (35%) female. The average age of the patients was 29 years. Thirteen diseases were noted in order to determine the causes of low vision in patients who participated in this study (Table 1 and Table 2 Causes of low vision). The results shows that about eighty one (81) patients were suffering from Optic atrophy which indicate that it is the main cause of low vision among the participating patients which makes up of 28.9% of the total patients. Retinitis pigmentosa was the second highest cause of prevalence of low vision, accounting for 73 patients that is 26% followed by Diabetic retinopathy and Macular degeneration which was found in 44 patients making up 15.7% and 16 patients making up 5.7% of the total population of the participants respectively.

**Table 1:** Causes of low vision

Causes	Number	Percentage
Optic Atrophy	81	28.9
Retinitis Pigmentosa	73	26
Diabetic Retinopathy	44	15.7
Macular Degenration	16	5.7
Refractive Error	16	5.7
Rod-Cone Dystrophy	8	2.9
Keratoconus	5	1.8
Congenital Nysatagmus	5	1.8
Albinism	6	2.1
Optic Neuropathy	3	1.1
Cataract	12	4.3
Retinal Detachment	3	1.1
Congenital Glaucoma	8	2.9
Total	280	100

**Table 2:** The most common ocular diseases causes low vision in different age group

Age Group	Causes of Low vision
Child patients	Optic Atrophy, Retinitis Pigmentosa, Congenital Glaucoma
Young patients	Refractive Error, Keratoconus, Albinism
Old patients	Diabetic Retinopathy, Macular Degenration, Cataract

Regarding their presenting visual acuity, around 149 patients had a visual acuity of 6/18 to 6/60 in the better eye; about 84 patients had a visual acuity between 6/60 to 2/60 in the better eye and 47 patients had visual acuity between 2/60 and counting fingers (CF) (Table 3 Presenting Distance Visual Acuity).

**Table 3:** Presenting Distance Visual Acuity

Presenting Visual Acuity (VA)	Number of patients
VA: 6/18 to 6/60 in the better eye	149
VA: 6/60 to 2/60	84
VA: between 2/60 and counting fingers (CF)	47

**Figure 1:**
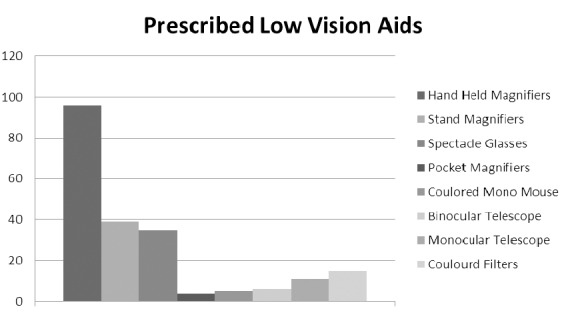
Low Vision Aids (LVA) given to patients with low vision

After examination of all the participants, low vision aids were prescribed to all of them. These devices were prescribed to the participating patients and the related information is as follows: 96 patients were given hand held magnifiers; about 39 were given fixed focus stand mounted magnifiers; 35 were give spectacle mounted glasses; 4 were given pocket magnifiers; 5 patients were given colored mono mouse; 6 patients were given binocular telescope; 11 patients were given monocular telescope and 15 participants were provided with colored filters. Improvement in visual acuity with low vision aids was ranged from 2 to 4 lines. There was no improvement in the visual acuity in 69 patients (Figure 1 Low Vision Aids (LVA) given to patients with low vision).

## 4. Discussion

A large number of those who would benefit from the low vision services may be denied the opportunity of doing so because of the increasing demand for the provision of these services (Silver, 1976). In terms of low vision causes, the result of this study shows that, the three main causes of low vision are Optic atrophy, retinitis pigmentosa and Diabetic retinopathy. Also, the study shows that, hereditary or congenital ocular diseases account for 65% of the cases. Theses ocular diseases are; Optic Atrophy, Optic Neuropathy, Retinitis Pigmentosa, Rod-Cone Dystrophy, Albinism, Congenital Nysatagmus and Congenital Glaucoma. These result also coincide with the results obtained by Al-Wadani et al study in 2012 (Al-Wadani et al., 2012). The exact cause of this is unknown, but it could be attributed to consanguineous marriage. The consanguinity rate in Saudi Arabia has been acknowledged to be as high as 56% and these hereditary or congenital conditions are not preventable. This may lead to an important issue in which a genetic counseling and clarification of the risks associated with birth defects among the children of directly related parents is also suggested (Al-Wadani et al., 2012). Consequently, a public awareness should be embarked on for the enlightenment on ocular diseases due to consanguineous marriage. On the other hand, Diabetic retinopathy accounts for 16% of low vision cases. These results are not surprising in which diabetic rate in adult in Saudi Arabia is high. The latest study shows that almost 1 in 11 people in Saudi Arabia have diabetes, and if the current prevalence rate remain constant over a long period of time, then the ratio of people with and devoid of diabetes will amplify astonishingly to almost 1 in 5 people by the year 2020 (Alhowaish, 2013; Alqurashi, Aljabri, & Bokhari, 2011).

A significant improvement in Visual acuity (VA) was noticed among low vision patients when Low Vision Aids (LVA) was used. These devices are very important in which it may bring them back and help them keep their independency as well as improving their quality of life. However, the LVA services are correspondingly in short supplies and very expensive if they want to buy it from a limited number of optical centers offering such aids in the country. At present, the LVA devices can only be given to patients on loan for a specific period of time. This practice of course does not encourage the patient’s continuous use of low vision aids knowing that once the low vision aids are returned and they would be left with nothing especially if they have no money to pay for again borrowing the low vision aids which implies they would be more handicapped. So that, a patient health care related to government and social societies should support and help those patients whom are in need of low vision aids.

According to the available and existing knowledge, most of the hospitals in Saudi Arabia do not have a low vision service even though, all of them reported how important it is to provide these services. Consequently, an attempt should be made from the Ministry of health to start establishing low vision clinics in major hospitals at first stage to close the gap between the number of low vision patients and number of low vision clinics and provide it with qualified specialists in low vision field.

## 5. Conclusion

Public awareness should be embarked on for enlightenment on ocular diseases due to consanguineous marriage. Also, it is an important issue to start establishing low vision clinics in order to meet the needs and requirements of predictable amplification in the number of low vision patients in the future, as population grows older. Hence on the basis of these results it can be concluded that the null hypothesis is correct and the other hypothesis is automatically eradicated.
